# A Comparative Evaluation of the Efficacy of Etching by the Total Etch and Self-etch Dentin Bonding Systems in the Primary Teeth: An *in vitro* Study

**DOI:** 10.5005/jp-journals-10005-1279

**Published:** 2015-04-28

**Authors:** Sajjad Hasim Mithiborwala, Vishwas Chaugule, Farhin Katge, Manohar Poojari, Prashant Pujari, Thejokrishna Pammi

**Affiliations:** Assistant Professor, Department of Pedodontics and Preventive Dentistry, Terna Dental College and Hospital, Navi Mumbai, Maharashtra, India; Professor and Head, Department of Pedodontics, Sinhgad Dental College, Pune Maharashtra, India; Professor and Head, Department of Pedodontics, Terna Dental College, Navi Mumbai, Maharashtra, India; Reader, Department of Pedodontics, Terna Dental College, Navi Mumbai, Maharashtra, India; Reader, Department of Orthodontics Dentistry, Pacific Dental College and Hospital, Udaipur, Rajasthan, India; Associate Professor, Department of Pedodontics, Terna Dental College, Navi Mumbai, Maharashtra, India

**Keywords:** Interfacial morphology, Total etch systems, Self-etch systems, Etching pattern.

## Abstract

**Objective:** Early childhood caries is now affecting the children in dangerous proportions. There is a wide spread loss of the tooth material irrespective of the type of the carious lesion. Restoration of such lesions with a strong permanent bond between the dental tissues and the restorative dental materials would be a highly desirable requisite. Ultramorphological characterizations show that the interfacial morphology and the chemical characterization of the bonding systems appear to be strongly associated with each other and, therefore, observing and understanding the interfacial phenomenon and its quality would be of great importance in the selection of a dental adhesive for its use in pediatric restorative dentistry.

**Study design:** Human primary molars, which were indicated for extraction, for an array of reasons like caries, normal exfoliation, pathological root resorption, over-retained and serial extraction, were collected for the study purpose. Total number of teeth was then equally distributed into two subgroups, each namely A1 (Prime and Bond NT) and A2 (Xeno III).

**Results:** The type of etching pattern that was observed in group A1 (Prime and Bond NT) of Silverstone’s type II compared to the Silverstone’s type III observed in group A2 (Xeno III).

**Conclusion:** Results of this study indicate that the use of an etchant separately followed by the application of the bonding system–Prime and Bond NT–would provide a better quality of adhesion thus improving the quality and longevity of the restoration done within the limits of enamel in primary dentition.

**How to cite this article:** Mithiborwala SH, Chaugule V, Katge F, Poojari M, Pujari P, Pammi T. A Comparative Evaluation of the Efficacy of Etching by the Total Etch and Self-etch Dentin Bonding Systems in the Primary Teeth: An *in vitro* Study. Int J Clin Pediatr Dent 2015;8(1):30-36.

## INTRODUCTION

Early childhood is marked by tremendous growth and development of the face and dentition, both of which require the attention of a dental professional. Among the more common oral conditions of early childhood, dental caries is the preeminent concern because of its tremendous prevalence and consequences. Overall, nearly one in five (18.7%) US children ages 2 to 4 have experienced visually evident tooth decay.^1^

National surveys conducted during the past three decades have demonstrated a decline in the overall mean levels of clinically detectable dental caries in US children and adolescents.^2^ Nevertheless, dental caries remains the single most common disease of childhood that is neither self-limiting nor amenable to short-term pharmacological management. More than 80% of the pediatric population is affected by dental caries by age 17. In a study conducted by Balwant Rai et al (2007), the mean DMFT was found to be 2.82, 2.87, 3.40, and 3.15 in 9 to 12 years old children while the mean DMFS was found to be 3.82, 3.87, 3.76, and 4.26.^3^

The operative decision is a significant one as the action is irreversible and restorations have a finite lifespan. Such a decision assumes that an active caries lesion is present and that no other more conservative therapy is possible to affect a successful outcome. Traditional cavity preparation includes varying degrees of ‘extension for prevention’ in an attempt to remove adjacent caries-prone tooth structure.

During the 32nd Annual Meeting of the International Association for Dental Research in 1954,^4^ Buonocore suggested that using 85% phosphoric acid solution resulted in an adhesion of acrylic resin to enamel.^5^ Similar to other conceptual and technologic innovations in the dental field, the etching procedure was introduced in dentistry ahead of its time and only 10 years later the bonding mechanism was described.^6^ After the introduction of acid etching procedure by Buonocore in 1954, the philosophy of mechanical retention of the restorations changed to micro-mechanical and chemical adhesion of the restoration to the tooth structure. Thus, the cavity preparation principles of GV Black’s ‘extension for prevention’ changed to a more conservative type, preserving the sound tooth structure with the understanding of adhesive dentistry.

Longevity of restorations is very low in the primary dentition.^7^ Generally, the earlier the age at restoration, the lower the longevity.^8,9^ The predicted life span of re-resto-rations is even shorter.^8^ The vast majority of clinical research on the primary dentition from the systematic review involves relatively short-term comparisons of dental materials, particularly newer proprietary materials, as they enter the marketplace. Qvist et al found that the major reasons for replacement of restorations in the primary dentition were restoration fracture or total loss of the same.^10^ There is a continuing search, to the present day, for improved materials as a restorative solution to caries management in the primary dentition.

Marginal leakage cannot be eliminated even when higher shear bond strength are obtained for some adhesive systems. Thus, it can be assumed that the magnitude of bond strengths is not the only predictor of the sealing ability. So, the development of bonding systems, which will provide a true and stable adhesive bond to tooth structure in the rigors of the oral environment, is a high priority.

The widespread demand for, and the use of dental adhesives have, thus, fueled an intense development of better and easier dental adhesives in rapid succession. The ‘generational’ definitions help in identification of chemistries involved, the strength of dentinal bond and the ease of use for the practitioner. The fundamental principle of adhesion or bonding to the tooth substrate is an exchange process where inorganic tooth material is exchanged for a synthetic resin. Though bonding to the enamel could be effectively achieved, bonding to dentin was a real challenge due to its heterogeneous nature and presence of water, presence of smear layer and smear plugs, etc. All these created a significant problem, because they prevented direct contact of dentin bonding agent to the dentin surface.

In a very short period of time single-bottle agents, acid primers and self-etching bonding systems became popular. However, these products should be evaluated and tested before they are used on a large-scale basis. Bonding systems have gone through a number of generational advancements, and overcome the early hiccups. Today, we have the bonding systems capable of providing predictable and durable bonding to both enamel and dentin with easy to use approaches.

Until recently all adhesive systems used in the past had three steps prior to restoration. These involved etching, priming and bonding. This was quite cumbersome. Hence, the thought process continued in the direction of reducing the number of steps involved in bonding prior to the restoration with better clinical results. Currently, there are two philosophies on simplification of the adhesive systems *viz*:

- The total etch systems with a separate etchant and a pr i mer/ad he sive.
- The self-etching systems which combine etching and priming in one bottle and have a separate adhesive agent or which combine all three steps in a single solution.

Studies comparing the total etch systems and self-etching systems showed results varying from no significant difference to higher or lower bond strength and sealing ability in primary dentition than in permanent dentition.^11,12^ Results of recent *in vitro* studies have revealed the lower efficacy of self etch system than the total etch system in primary dentition. Chemical, physiological and micro morphological differences, such as decreased mineralization, small size and lower concentration of dentinal tubules, decreased permeability and more reactivity of primary dentin to acidic conditioner were thought to be responsible for lower bond strength and sealing ability in primary dentition.^13^ Despite simpli-fication of bonding systems, technique sensitivity and substrate variability; concerns about enamel and dentin bonds have increased.

In the light of these developments, this study is undertaken to compare the behavior of both the 5th generation total etch bonding system (Prime and Bond NT) and the 6th generation self etch bonding system (Xeno III) by examining their etching pattern in primary teeth on the basis of the interfacial morphology.

## MATERIALS USED IN THE STUDY

- Bonding agents: Prime and Bond NT (LOT–051123 2007-11) and Xeno III (LOT – 0605000856-857 2008-04) bonding adhesives.
- HILUX dental curi ng l ig ht–Ku l zer, Ben lioglu Dental Inc., Turkey. Order no–950-200-230, Class II equipment.
- Gold palladium sputtering unit: JEOL JFC-1600 Auto Fine Coater, Tokyo, Japan.
- Analytical scanning electron microscope–JSM-6360A, JSM-6360LA.
- Double-sided diamond disks procured from Dental World, Pune.
- 0.5% chloramine T powder.

## METHODOLOGY

Twenty-four human primary molars, which were indicated for extraction, for a variety of reasons like caries, normal exfoliation, pathological root resorption, over-retained and serial extraction, were collected for the study purpose. They were stored in 0.5% chloramine T solution at 37°C until further procedures.^14^

Total number of teeth was then equally distributed into two subgroups, each namely group A1–Prime and Bond NT and group A2–Xeno III.

### Inclusion Criteria for Teeth Selection

Since the thickness of enamel is found to be the greatest on the buccal surface followed by lingual surface, one of these surfaces was chosen for studying the etching pattern.^15,16^ Selection of intact buccal or lingual surfaces, which were devoid of any intrinsic stains, developmental anomalies and developmental abnormalities, was done.

**Fig. 1:**
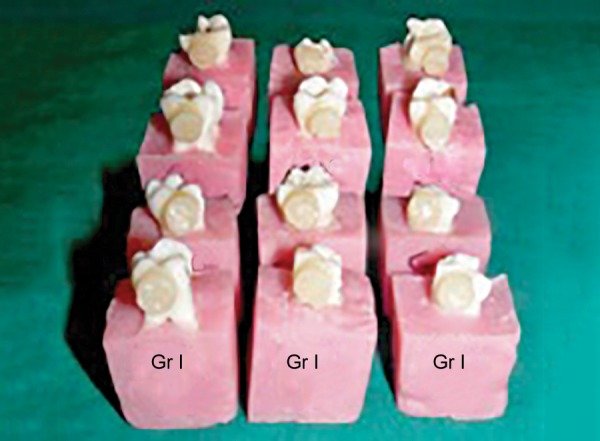
Composite cylinders built-on prepared specimens in samples of group A1

**Fig. 2:**
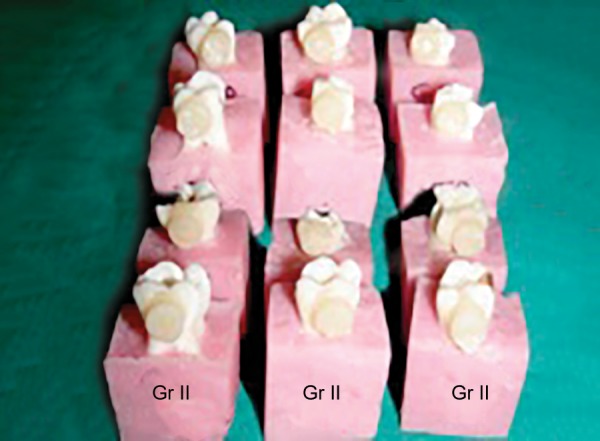
Composite cylinders built-on prepared specimens in samples of group A2

### Procedure^14^

**Fig. 3:**
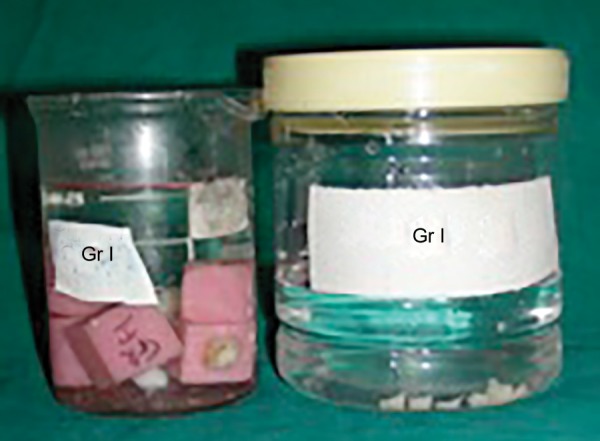
Decalcification procedure in samples of group A1

**Fig. 4:**
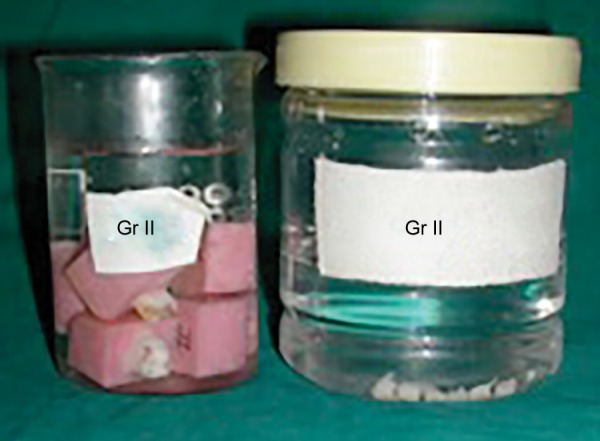
Decalcification procedure in samples of group A2

At the outset of the procedure teeth were mounted in the blocks prepared out of pink self-cured acrylic resin using L-molds. Either buccal or lingual enamel surface of the specimens, which fulfilled the inclusion criteria, was fattened using a diamond disk. Care was taken not to expose the dentin. The prepared surfaces were first cleaned with RC Prep for 1 minute to remove the inorganic debris^17^ and then rinsed thoroughly with 3% sodium hypochlorite using 5 cc disposable syringe with 24-gauge needle to remove the organic debris.^18,19^ Teeth were air-dried of all the visible moisture with a moderate short blast of air.^20^ Bonding procedure for both the groups was carried out according to manufacturer’s instructions. In order to obtain a negative replica of the etch pattern of the bonded specimen, the cylinders of packable composite^14^ were fabricated over the bonded area with the help of a straw having the diameter of 7 mm and the height of approximately 5 mm. The composite cylinders were cured in increments till the height of cut straw (Figs 1 and 2). Bonded teeth were then immersed into a beaker containing 5 % concentrated nitric acid at room temperature and observed for 24 to 36 hours for enamel decalcification 14 (Figs 3 and 4). Most of the composite cylinders got detached within a period of 18 to 20 hours whilst the remaining ones got separated before the stipulated time. The impression surface of all the composite cylinders was thoroughly cleaned with distilled water using 5 cc syringe with 24-gauge needle. The specimens were air-dried for 24 hours by keeping them uncovered in a tray for the purpose of sputtering prior to subjecting them for SEM examination.

## RESULTS

Only a few representative electron photomicrographs have been included as the total number of photomicrographs was quite large.

### Photomicrographs showing the Etching Pattern on Primary Enamel in Group A1 (Prime and Bond NT)

**Fig. 5:**
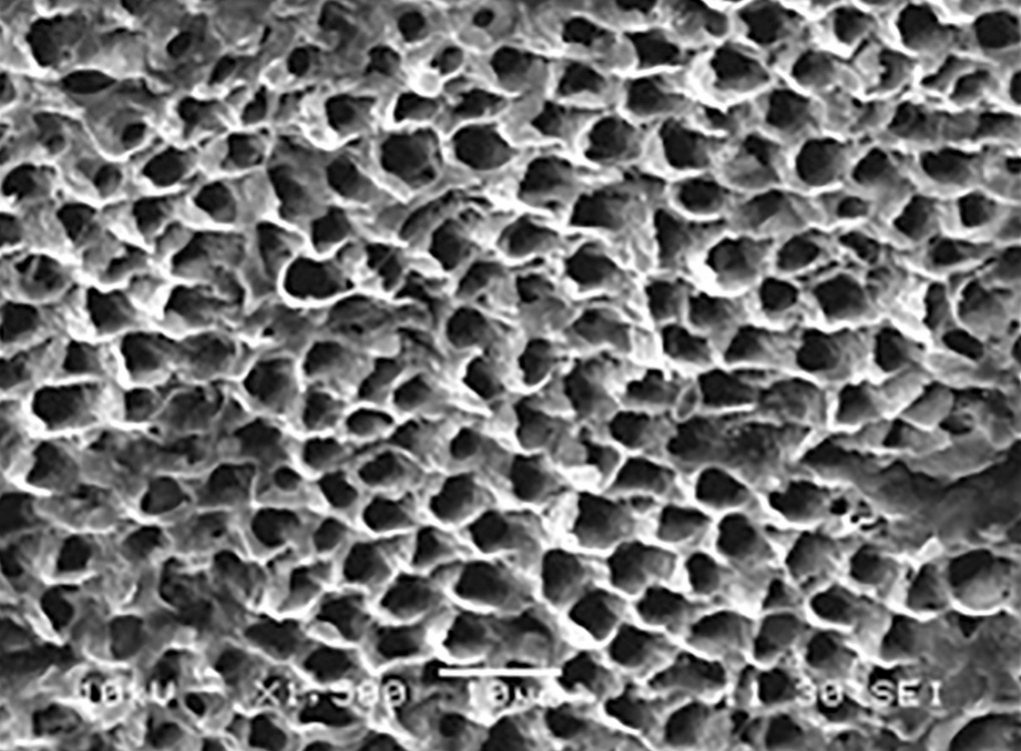
Type II etching pattern in group A1 at 1500×

Figure 5 shows the etching pattern as a reverse honeycomb pattern, seen on the prepared enamel surface. Hence, the pattern seen in Figure 1 of this study is a Silverstone’s type II etching pattern as seen at 1500× magnification.

**Fig. 6:**
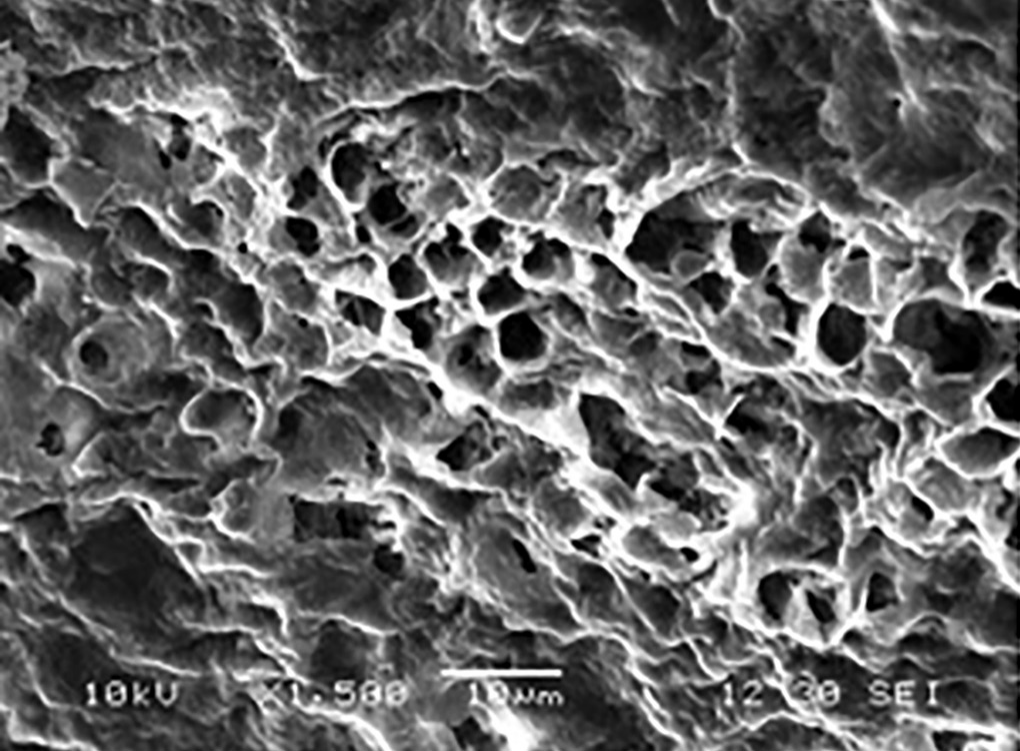
Mixed type of etching pattern in group A1 at 1500×

**Fig. 7:**
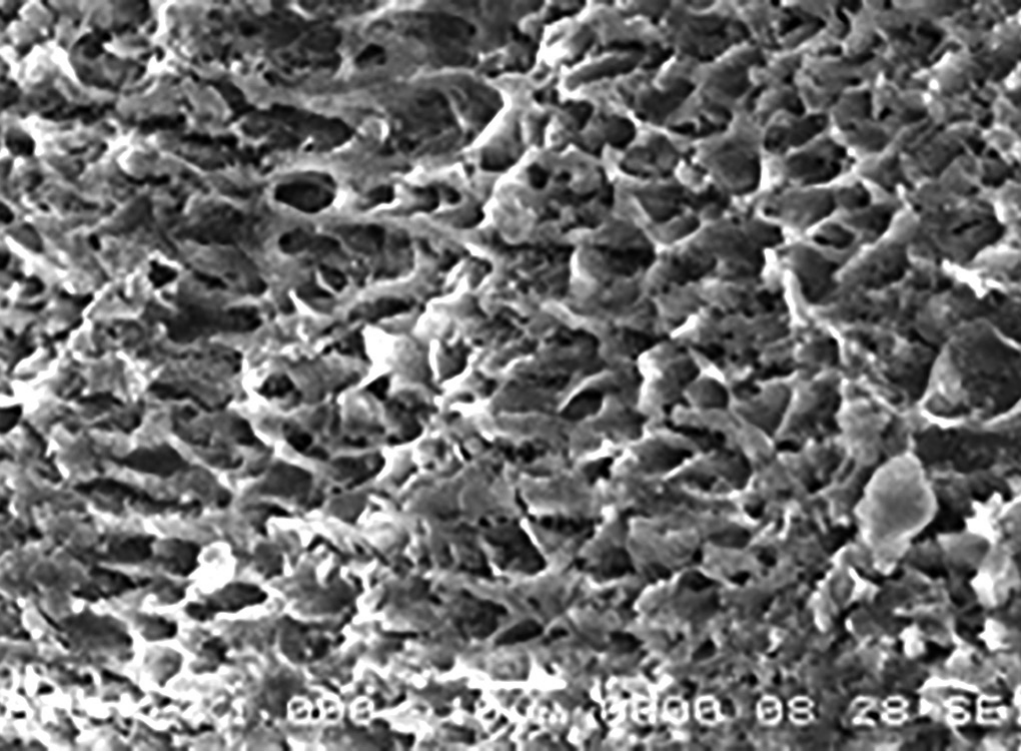
Type III etching pattern in group A2 at 1000×

**Fig. 8:**
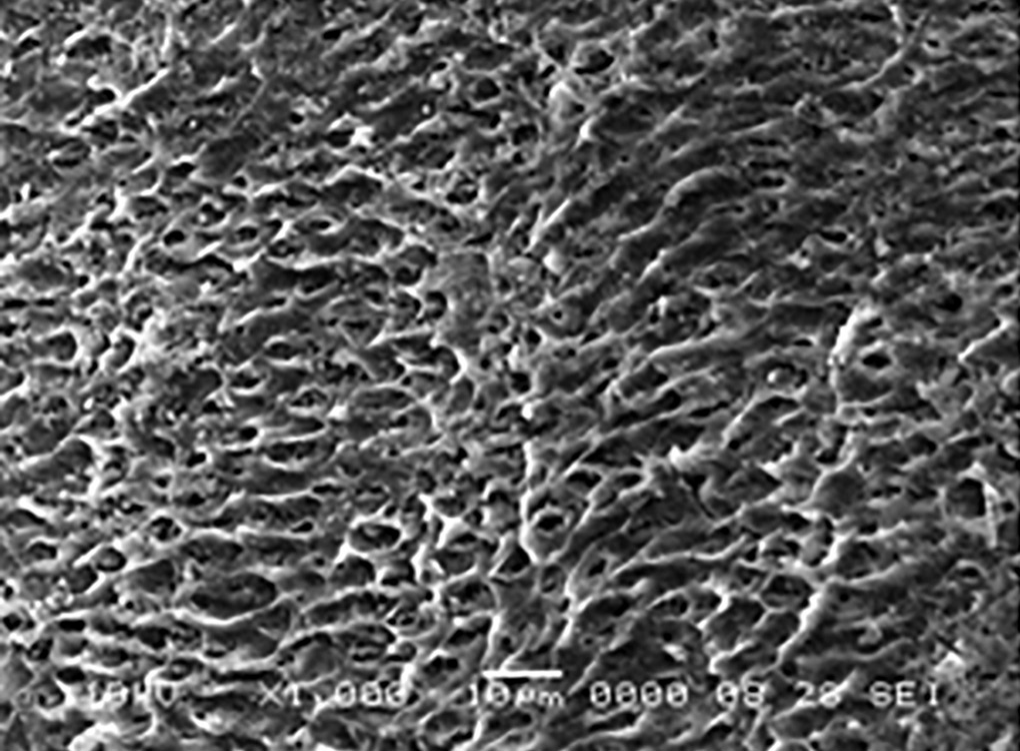
Mixed type of etching pattern in group A2 at 1000×

Figure 6 shows mixed type of etching pattern noticed in one of the samples from the same group, may be due to different orientation of enamel rods on the same surface, thus, creating different microregions of etching pattern.

### Photomicrographs showing the Etching Pattern on Primary Enamel in Group A2 (Xeno III)

Figure 7 shows type III etching pattern in the samples of group A2 (Xeno III).

Mixed pattern of etching is visible in the Figure 8 when one of the samples of group A2 (Xeno III) was examined under SEM.

### Etching Pattern in Group A1 (Prime and Bond NT)

**Table 1:** **Table 1:** The presence or absence of different types of etching patterns seen in the samples of group A1 (Prime and Bond NT)

*Sample*		*Etching pattern*							
*no.*		*Type I*		*Type II*		*Type III*		*Mixed*	
1.		Absent		Present		Absent		Absent	
2.		Absent		Present		Absent		Absent	
3.		Absent		Present		Absent		Absent	
4.		Absent		Present		Absent		Absent	
5.		Absent		Present		Absent		Absent	
6.		Absent		Present		Absent		Absent	
7.		Absent		Present		Absent		Absent	
8.		Absent		Present		Absent		Absent	
9.		Absent		Present		Absent		Absent	
10.		Absent		Present		Absent		Absent	
11.		Absent		Present		Absent		Absent	
12.		Absent		Absent		Absent		Present	

### Etching Pattern in Group A2 (Xeno III)

**Table 2:** **Table 2:** The various etching patterns seen in group A2 (Xeno III)

*Sample*		*Etching pattern*							
*no.*		*Type I*		*Type II*		*Type III*		*Mixed*	
1.		Absent		Absent		Present		Absent	
2.		Absent		Absent		Present		Absent	
3.		Absent		Absent		Absent		Present	
4.		Absent		Absent		Present		Absent	
5.		Absent		Absent		Absent		Present	
6.		Absent		Absent		Present		Absent	
7.		Absent		Absent		Absent		Present	
8.		Absent		Absent		Absent		Present	
9.		Absent		Absent		Absent		Present	
10.		Absent		Absent		Absent		Present	
11.		Absent		Present		Absent		Absent	
12.		Absent		Absent		Present		Absent	

## STATISTICAL ANALYSIS

A statistical analysis and computation procedure were performed. Data collected were entered into MS-Excel worksheet and use of ‘Statistical Package for Social Sciences’ (SPSS) software was used. Results were represented in the form of tables (Tables 1 and 2) and graphs.

The results were expressed in the form of percentage. To observe and assess the etching pattern in primary teeth, ‘Z test of proportion’ was applied to determine the type of etching pattern.

p-value was determined at 95% confidence limits for all the tests mentioned above.

## STATISTICAL RESULTS

**Table 3:** **Table 3:** The statistical analysis and the p-value

*Etching pattern*		*Prime and Bond NT*		*Xeno III*		*p-value*		*Statistically significant*	
Type II		11		1		0*		Yes	
Type III		0		5		-*		Yes	
Mixed		1		6		0.07		NS	

*p < 0.01 (highly significant); NS: Not significant; Z: 3.678; p = 0.000 (p < 0.05)

Since the p-value is less than 0.01, the difference is highly significant statistically when we compare the type II etching pattern in both the groups.

From the results of Table 3, it is seen that:

- Type I etching pattern is not present in either of the groups.
- Type II etching pattern is more commonly seen in group A1 as compared to that of group A2.
- Type III etching pattern is more commonly seen in group A2 as compared to that of group A1.
- Mixed etching pattern does not show any significant statistical difference.

**Graph 1:**
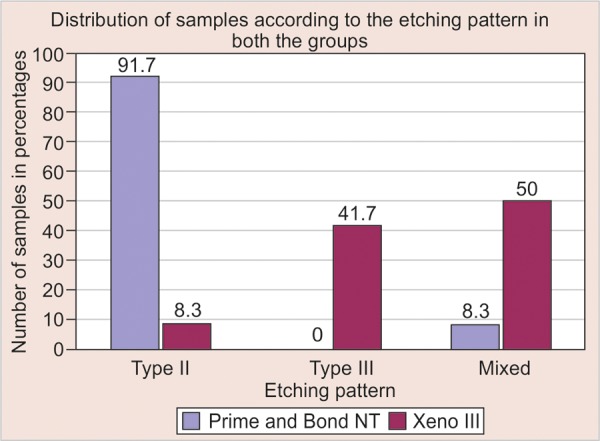
Occurrence of the etching patterns in percentages in both the groups

Graph 1 is a bar diagram which represents the occurrence of the etching patterns in percentages in both the groups. The group A1 (Prime and Bond NT) shows 91.7 and 8.3% of occurrence of type II and mixed etching pattern respectively, whereas the group A2 (Xeno III) shows 8.3, 41.7 and 50% of occurrence of type II, type III and mixed etching patterns respectively.

## DISCUSSION

There is a plethora of information available on the mechanism of adhesion for bonding systems on permanent teeth. Primary teeth are smaller in size, have thinner enamel and dentin, and show a rapid spread of dental caries. Hence, with less tooth structure available for bonding of composite resin material, proper dentin bonding steps should be followed for success of the composite restoration in primary teeth. Achievement of a consistently reliable, gap-free and complete attachment of resin composite to dentin is of profound importance in restorative dentistry. Formation of an acid resistant, resin impregnated hybrid layer seems to depend on the penetrating ability of resin into etched dentin surface and also on conditioning and permeability of dentinal surface.^23,24^ However, the similar type of information on primary teeth appears to be scanty. Few studies in the past stated the presence of formation of thicker hybrid layers^25^ in primary teeth with shorter resin tags.

The results of the etching pattern in this study showed the type II etching pattern to be seen more commonly in group A1 (Prime and Bond NT) as compared to the type III etching pattern seen in group A2 (Xeno III).

In the study, the results for group A1 (Prime and Bond NT) were in accordance to the ones observed by Hosoya^26^ (1991) who stated that the Silverstone’s type II pattern was the most prevalent in deciduous teeth, despite the time employed, the region etched and the depth of the enamel. He also speculated that 10 seconds and 20 seconds acid conditioning would produce unsatisfactory alterations.

Prism cores and boundaries etched by 34 and 35% phosphoric acid, caused dissolution of both inter and intraprismatic enamel areas and found that the predominant etching pattern was type II. They also stated that the acid etching with phosphoric acid was found to be superior and it provided good adhesion to both ground and ungrounded enamel surface.^21,27^

However, the results obtained after using Xeno III (group A2) in our study were different. The etching pattern observed in group A2 (Xeno III) was Silverstone’s type III affirms that the self-etching primers produce a more conservative etch pattern than phosphoric acid.

Silverstone et al demonstrated that there was a tendency to predominating nonprismatic or type III pattern with 15s, 60 to 90 seconds etching time on human deciduous enamel surfaces.^21^ From a clinical standpoint, the use of self-etching primers could be desirable because they reduced clinical steps, saved chair time, improved the adhesive procedures and reduced the risk of salivary contamination.^28^

The observation of the mixed etching pattern found in both the groups in this study led us to conclude that at the moment of enamel instrumentation, the prisms are exposed in several planes according to their direction. This difference in the angulations of the prism crystals causes the acid to have higher demineralization potential at certain micro regions, which results in different acid-etching patterns.^29^

Compiling the observations of the present study, it implies that the demineralization by acid etching is selective because of morphological disposition of the enamel rods and the prisms, and it may play an important role in determining the type of etching pattern seen in primary aprismatic enamel rather than the concentration of the acid etchant and the etching time. Since the reduction in the technique sensitivity of any bonding system would always be a preferred factor in pediatric restorative dentistry, further studies should be carried out keeping in mind the above variables toward the development of a universal bonding system. Therefore, after having gone through the review of literature and the study conducted with its limitations, it appears that the inclination towards the selection of the bonding system may lean towards the self-etching bonding system. The candid recommendation for the use of any specific bonding system in pediatric dentistry seemed to be difficult at this juncture due to the availability of the limited resources at the time of conducting the study.

## CONCLUSION

The etching pattern study led us to conclude that the type of etching pattern that was observed in group A1 (Prime and Bond NT) was of Silverstone’s type II^21,22^ compared to the Silverstone’s type III observed in group A2 (Xeno III). Thus, the etching pattern in the total etch group A1 (Prime and Bond NT) was found to be superior to that of group A2 (Xeno III).

### What This Paper Adds

Results of this study indicate that the use of an etchant separately followed by the application of the bonding system–Prime and Bond NT–would provide a better quality of adhesion, thus, improving the quality and longevity of the restoration done within the limits of enamel in primary dentition.

### Importance to a Pediatric Dentist

Despite the micromorphological and histological difference in primary teeth, manufacturers do not provide separate specific instructions for the use of dental adhe-sives in primary dentition. So, the pediatric dentist has to be selective while choosing the appropriate bonding system for its use in pediatric dentistry.
